# Sub-Nanogram Detection of RDX Explosive by Monoclonal Antibodies

**DOI:** 10.1089/mab.2015.0010

**Published:** 2015-08-01

**Authors:** David O. Ulaeto, Alistair P. Hutchinson, Stephen Nicklin

**Affiliations:** ^1^ Biomedical Sciences Department, Dstl Porton Down, Salisbury, United Kingdom.; ^2^ Detection Department, Dstl Porton Down, Salisbury, United Kingdom.

## Abstract

Polyclonal and monoclonal antibodies were raised to protein carrier molecules haptenized with RDX, a major component of many plastic explosives including Semtex. Sera from immunized mice detected RDX protein conjugates in standard ELISA. Clonally purified monoclonal antibodies had detection limits in the sub-ng/mL range for underivatized RDX in competition ELISA. The monoclonal antibodies are not dependent on the presence of taggants added during the manufacturing process, and are likely to have utility in the detection of any explosive containing RDX, or RDX contamination of environmental sites.

## Introduction

RDX is an explosive chemical^(1)^ that is one of the major components of plastic explosives including Semtex. A high affinity monoclonal antibody specifically recognizing it would have useful properties in the detection of trace amounts of RDX-based explosives and related compounds. RDX is a reactive hapten that, because of its small size, is unable to directly crosslink immunoglobulin molecules on the surface of RDX-specific B cells. Because it is not a protein, it is also unable to stimulate T cell help.^(2)^ In order to generate a high affinity antibody to RDX, it is necessary to chemically conjugate the hapten to a large protein molecule that is capable of eliciting T cell help.

Before the hapten can be conjugated to a protein it must be chemically derivatized to enable it to bind covalently to the protein molecule.^(3,4)^ This process alters the structure of the hapten, raising the possibility that an antibody that recognizes the derivatized hapten may not in fact recognize underivatized pure RDX. In addition, some derivatives possess a relatively long side chain that is not found in the underivatized hapten. Because of the small size of the hapten itself, antibodies raised to the derivative may include portions of the derivative side chain as recognition features, leading to a failure to recognize the hapten with sufficiently high affinity in the absence of the side chain.

## Materials and Methods

**FIG. 1.**
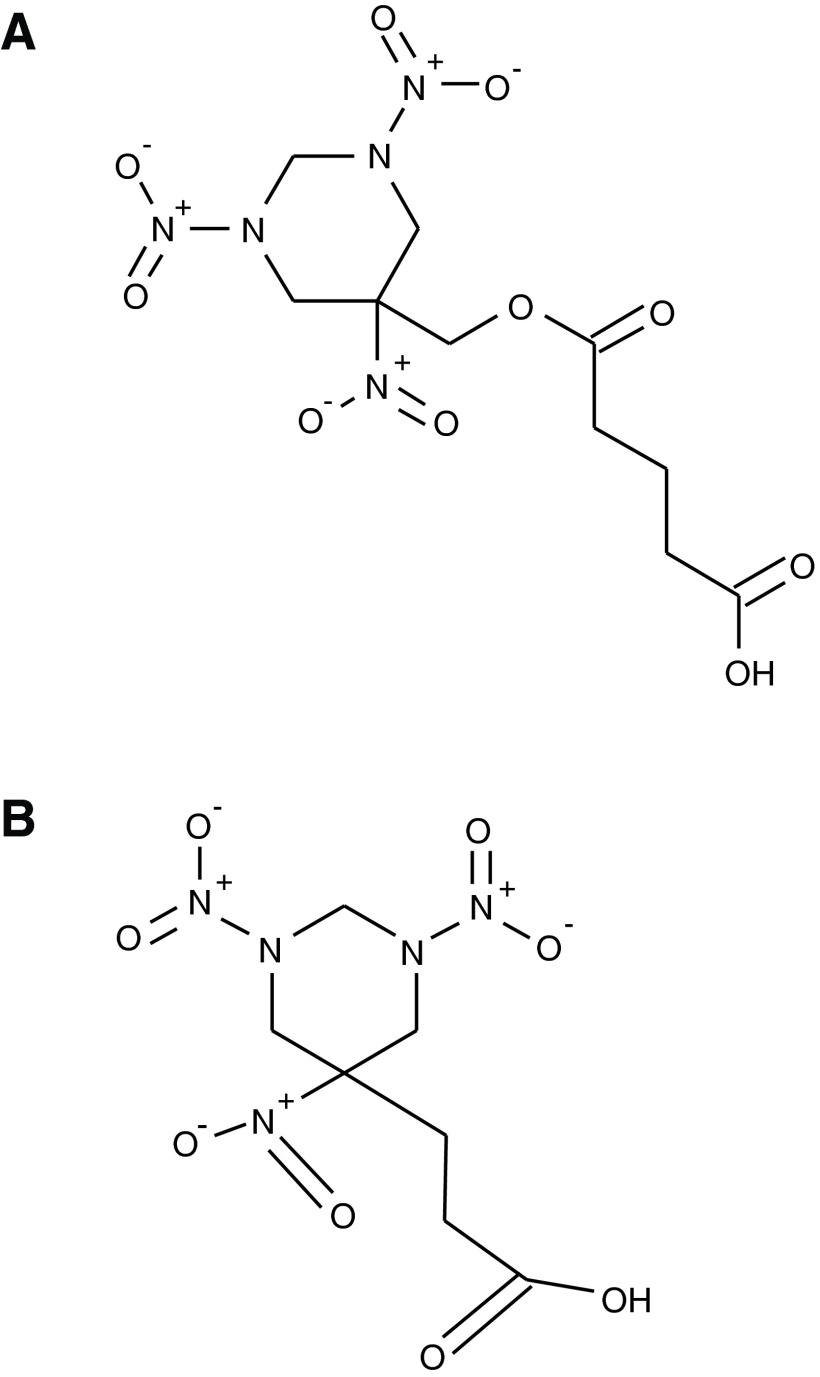
Structure of RDX12 (**A**) and RDX14 (**B**). RDX and RDX derivatized haptens were synthesized as previously described.^(7)^ Derivatized haptens were conjugated to carrier proteins as follows: 65 mg EDC-1-ethyl-3-(3-dimethylaminopropyl) carbodiimide and 75 mg SULFONHS 1-hydroxy-2-5-dioxo-3 pyrrolidine sulfonic acid monosodium salt were added to 16.51 mg of RDX hapten dissolved in 1 mL of 50% N,N-dimethyl formaldehyde (v/v) in water. The mixture was stirred at room temperature for 30 min. After mixing, 20 mg of BSA, KLH, or CGG, respectively, was dissolved in 2 mL water and added to the mixture, which was then stirred for a further 3 h at room temperature. Each conjugate was collected and dialyzed extensively against four 3 L changes of PBS. Animal studies were performed in accordance with the UK Scientific Procedures Act (Animals) 1986 and UK Codes of Practice for the Housing and Care of Animals Used in Scientific Procedures (1989).

To avoid the above scenarios, two different derivatives of RDX were used in this project, RDX12, which has a relatively long derivative side chain, and RDX14, which has a very short derivative side chain (Fig. 1). RDX14 conjugates were used for immunizations of 5- to 8-week-old female BALB/c mice, and an RDX12 conjugate was used for screening of sera and hybridoma supernatants. The rationale for this is that only antibodies reactive with both derivatives will be selected, and the only features in common between the derivatives are those that are also shared by underivatized RDX. To further optimize the protocol, the derivatized haptens were conjugated to different carrier proteins, keyhole limpet hemocyanin (KLH) and bovine serum albumin (BSA), to ensure that carrier protein determinants were not part of the epitopes recognized by expanded antibodies. KLH-RDX14 conjugate emulsified in complete Freund's adjuvant (CFA) was used for all primary immunizations; but the mice were also simultaneously immunized with unconjugated BSA, emulsified in CFA, from a separate syringe at a separate site. Booster immunizations used a BSA-RDX14 conjugate emulsified in incomplete Freund's adjuvant. Hybridomas were generated by standard techniques using the X63-AG8 plasmacytoma fusion partner.^(2,5)^

## Results and Discussion

**FIG. 2.**
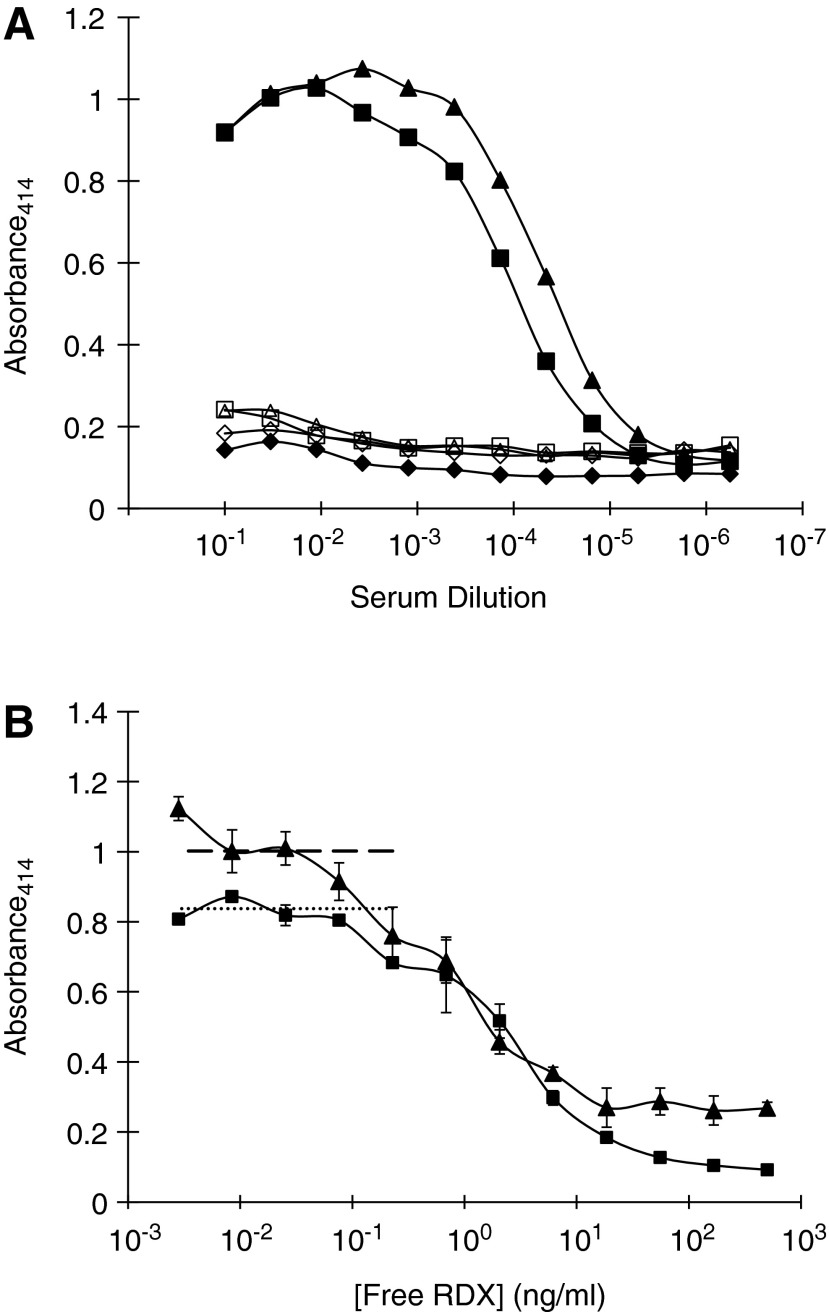
(**A**) Direct ELISA of IND (■, □) and SP (▲, ▵) RDX-immune mouse sera, and control normal mouse serum (NMS) (♦, ⋄) against CGG-RDX12. CGG-RDX12 (solid symbols) and unconjugated CGG (open symbols) were non-specifically adsorbed to Immulon-2 ELISA plates (Dynatech) at 5 μg/mL in carbonate buffer (pH 9.5) (Sigma). After blocking excess binding capacity of plates with gelatin at 1 mg/mL in carbonate buffer, sera were titrated across the plates in serial dilutions of 1:3 in PBS, starting with 1:10 dilution. Specifically bound antibody was detected with horse-radish peroxidase-conjugated goat anti-mouse antibody (Bio-Rad) in conjunction with an ABTS color development system (Sigma), by absorbance at 414 nm. (**B**) Competition of hybridoma supernatant binding to CGG-RDX12 by pure RDX. CGG-RDX12 was non-specifically adsorbed to ELISA plates, as described in Figure 1A. In a second plate, also blocked with gelatin, a solution of pure RDX in methanol was serially diluted in methanol in steps of 1:3, after which culture supernatant from Mall (▲) or Mazi (■) anti-RDX hybridomas was added to appropriate wells at a predetermined dilution. Half of the material (100 μL) from each well was transferred to the corresponding well on the CGG-RDX12-coated plate and a standard ELISA protocol followed, as described in Figure 1A. Data are presented as means and standard deviations of triplicate samples. Baselines were established as means of 12 wells for each hybridoma supernatant in the presence of methanol with no RDX (Mall, dashed line; Mazi, dotted line). One representative of multiple experiments.

Initial screens of sera and hybridoma supernatants were undertaken by direct ELISA against RDX12 conjugated to chicken gamma globulin (CGG). CGG was used as a carrier protein as a further precaution against selection of antibodies for which the carrier protein contributed to the recognized epitope. Serum from immunized animals was able to recognize CGG-RDX12 in direct ELISA at dilutions in excess of 1:1000 (Fig. 2A), indicating that the immunization was successful and hybridoma fusions could be undertaken with a high probability of success. After selection and expansion of hybridomas reactive with CGG-RDX12, further screening was undertaken using competition ELISA where recognition of CGG-RDX12 was competed with underivatized, unconjugated RDX. RDX efficiently competed with CGG-RDX12 for antibody binding, demonstrating specificity for RDX rather than a fortuitous epitope involving carrier protein or derivatization side chain moieties. The limit of detection for RDX was as low as 0.3 ng/mL for the best performing antibodies (Fig. 2B).

Since the early 1990s, it has been an international requirement for explosives to include a volatile taggant that is added during manufacturing.^(6)^ These make a major contribution to detection of bombs, landmines, and explosive caches by sniffer dogs and machine-based detection systems. The antibodies we describe here provide a useful complementary tool to the use of taggants, because they are able to directly and specifically bind RDX, irrespective of the presence of taggant. This has the potential to allow detection and identification of trace amounts of RDX-containing explosives for screening, forensic, or environmental analysis. Several technologies are available for the incorporation of antibodies into optical- and/or resonance-based sensors, which can import the sensitivity and specificity of the antibodies into extant and future sensor and detection technology.
